# No Direct Postconditioning Effect of Poloxamer 188 on Mitochondrial Function after Ischemia Reperfusion Injury in Rat Isolated Hearts

**DOI:** 10.3390/ijms22094879

**Published:** 2021-05-05

**Authors:** Josephine Eskaf, William J. Cleveland, Matthias L. Riess

**Affiliations:** 1 Department of Anesthesiology, Vanderbilt University Medical Center, Nashville, TN 37232, USA; josi.eskaf@gmail.com (J.E.); will.j.cleveland@gmail.com (W.J.C.); 2 Department of Anesthesiology, University Medicine Greifswald, 17475 Greifswald, Germany; 3 Department of Pharmacology, Vanderbilt University, Nashville, TN 37232, USA; 4 Anesthesiology, TVHS VA Medical Center, Nashville, TN 37212, USA

**Keywords:** ATP synthesis, calcium retention capacity, cardiac, cardiac arrest, copolymer, mitochondria, myocardial infarction, oxygen consumption, P188, PEG

## Abstract

Myocardial infarction is a leading cause for morbidity and mortality worldwide. The only viable treatment for the ischemic insult is timely reperfusion, which further exacerbates myocardial injury. Maintaining mitochondrial function is crucial in preserving cardiomyocyte function in ischemia reperfusion (IR) injury. Poloxamer (P) 188 has been shown to improve cardiac IR injury by improving cellular and mitochondrial function. The aim of this study was to show if P188 postconditioning has direct protective effects on mitochondrial function in the heart. Langendorff prepared rat hearts were subjected to IR injury ex-vivo and reperfused for 10 min with 1 mM P188 vs. vehicle. Cardiac mitochondria were isolated with 1 mM P188 vs. 1 mM polyethylene glycol (PEG) vs. vehicle by differential centrifugation. Mitochondrial function was assessed by adenosine triphosphate synthesis, oxygen consumption, and calcium retention capacity. Mitochondrial function decreased significantly after ischemia and showed mild improvement with reperfusion. P188 did not improve mitochondrial function in the ex-vivo heart, and neither further P188 nor PEG induced direct mitochondrial protection after IR injury in this model.

## 1. Introduction

Myocardial infarction (MI) is the leading cause for morbidity and mortality worldwide [1]. Without prompt reperfusion, the ischemic insult will lead to irreversible damage and long-term complications. However, reperfusion itself presents with a paradoxical exacerbation of tissue injury and cellular dysfunction [2]. This is known as ischemia-reperfusion (IR) injury. Outcomes after MI have improved in high-income countries in the last decades, mostly due to the reduction of time to reperfusion [1,3], but current guidelines and clinical practice still lack treatment of the secondary injury caused by reperfusion.

Mitochondrial dysfunction is a central part of IR injury [4]. Oxygen (O_2_) depletion during ischemia stops mitochondrial adenosine triphosphate (ATP) synthesis by inhibiting the electron transport chain (ETC) [5]. In an attempt to salvage mitochondrial membrane potential, the ATP synthase reverses function and starts hydrolyzing glycolytically produced ATP, further depleting the cell of energy [6].

The mitochondrial permeability transition pore (mPTP) is a non-specific voltage-dependent channel in the inner mitochondrial membrane (IMM) that is triggered to open upon high mitochondrial Ca^2+^-concentration and leads to an abrupt increase in IMM permeability [7,8]. This is a major determinant for the extent of injury during myocardial IR injury [7].

Uncoupling of the respiratory chain during ischemia also leads to enhanced production of reactive oxygen species (ROS). In a vicious cycle, ROS promote further damage to the respiratory chain, as well as Ca^2+^-overload [9].

The amphiphilic triblock copolymer Poloxamer (P) 188 (8400 Da) has been shown to improve myocardial function after IR [10,11,12,13,14,15,16] by acting as a patch on damaged membranes and restoring membrane integrity [17]. More recently, studies found P188 to improve mitochondrial function in in-vitro neurons [18,19], a porcine model of myocardial infarction [16], and it has been shown to be active in mitochondrial membranes in neurons [18].

Therefore, we hypothesized that:

(1) P188 improves mitochondrial function when given immediately upon reperfusion in rat isolated hearts.
(2) P188 improves mitochondrial function when given during isolation of mitochondria after IR injury in rat isolated hearts.
(3) The effect of P188 is not purely osmotic, but depends on its hydrophobic portion and cannot be substituted with the completely hydrophilic molecule polyethylene glycol (PEG, 8000 Da).

## 2. Results

### 2.1. Myocardial Function in the Ex-Vivo Heart

**Figure 1:**
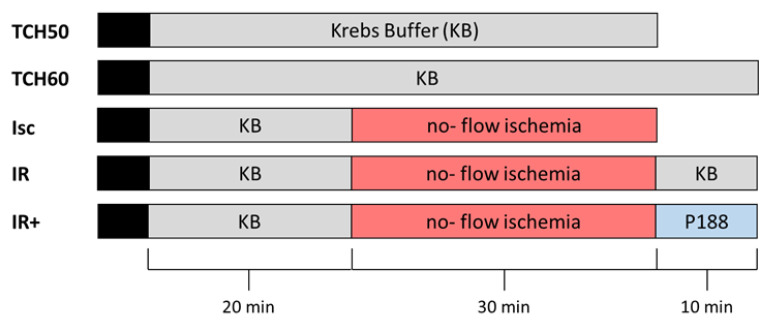
Timeline of experimental protocol in the different groups. Colors represent treatment of the heart. Black—preparation of the isolated heart; grey—perfusion with Krebs buffer (KB); red—no-flow ischemia; blue—perfusion with 1 mM Poloxamer (P) 188 in KB. TCH—time control heart; Isc—ischemia; IR—ischemia reperfusion; IR+—IR with 1 mM P188 during reperfusion.

Hearts were randomized into 5 groups (Figure 1). All hearts were perfused with Krebs buffer (KB) for 20 min before being subjected to 30 min of no-flow ischemia (Isc), prior to reperfusion for 10 min with KB (IR) or 1 mM P188 in KB (IR+). Two time controls (TCH 50, TCH 60) were perfused with KB for 50 and 60 min, respectively.

**Figure A1:**
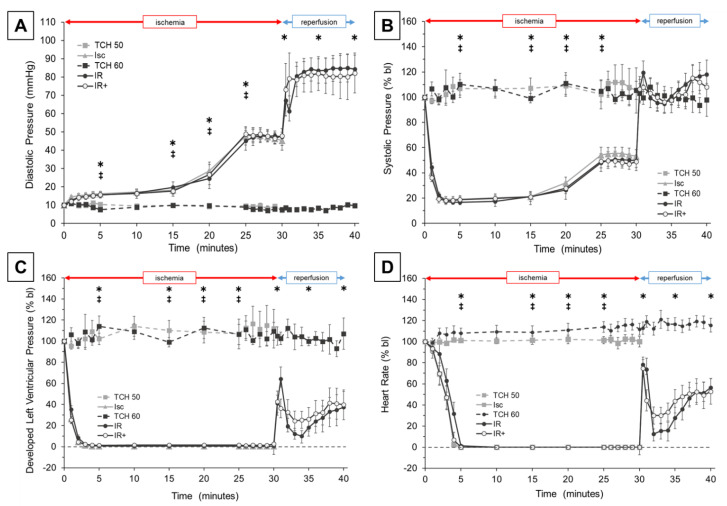
Myocardial function over time in rat isolated hearts during ischemia (Isc; *n* = 9) and reperfusion (IR; *n* = 9), compared to IR with P188 post-conditioning (IR+; *n* = 10) and time controls perfused for 50 or 60 min (TCH 50, TCH 60; each with *n* = 8) with Krebs buffer. For easier comprehension, non-parametric data are presented as mean ± SEM. Statistically significant difference (*p* < 0.05), as tested with Kruskal Wallis testing and post-hoc comparison by Dunn’s test, is indicated by ‡ (TCH 50/Isc), * (TCH 60/IR/IR+). Of note, there was no statistically significant difference between IR and IR+ at any time. (**A**) Change in diastolic pressure, normalized to 10 mmHg at baseline (bl). (**B**) Change in systolic pressure, represented as % bl. (**C**) Change in developed left ventricular pressure (devLVP = systolic pressure—diastolic pressure), represented as % bl. (**D**) Change in heart rate, represented as % bl.

Functional data of the heart were collected ex-vivo in the isolated heart during stabilization, ischemic, and reperfusion intervals. End points collected include: diastolic and systolic pressure, developed left ventricular pressure (devLVP, systolic pressure—diastolic pressure) and heart rate (HR). Except for diastolic pressure, all data are shown as percentage of baseline (% bl). The corresponding figures can be found in the appendix (Figure A1).

In all data collected, time controls (TCH 50, TCH 60) are analogous to each other and their function was steady over the 50 or 60 min duration of the experiment, respectively. Myocardial function decreased during the ischemic interval and showed significant damage in Isc, IR, and IR+ equally. Reperfusion slightly improved the function of the myocardium in IR and IR+ but 10 min of P188 post-conditioning showed no further improvement of myocardial function compared to no treatment.

### 2.2. Mitochondrial Function

Cardiac mitochondria from all ex-vivo groups were isolated with isolation buffer (IB; no treatment) or treated with 1 mM P188 or 1 mM PEG in IB, respectively.

#### 2.2.1. ATP Synthesis

**Figure 2:**
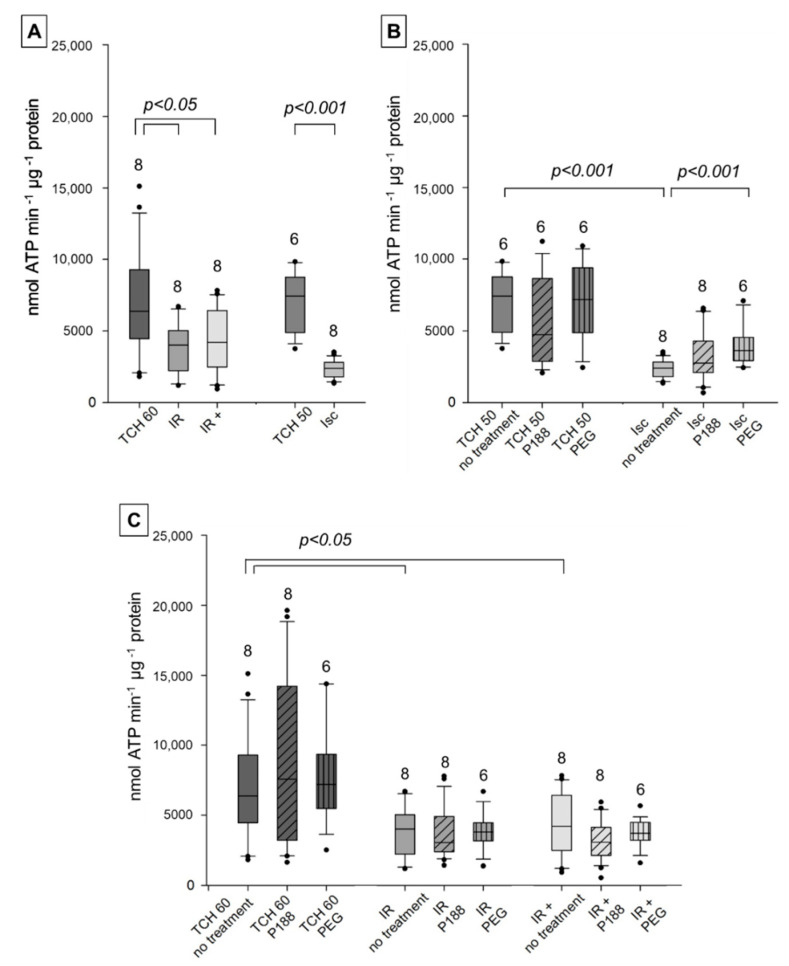
Mitochondrial adenosine triphosphate (ATP) synthesis (nmol ATP min^−1^ μg^−1^ protein) with complex I substrates in isolated cardiac mitochondria. Mitochondria isolated from rat isolated hearts subjected to ischemia (Isc), ischemia reperfusion (IR), IR with P188 during reperfusion (IR+) and only perfusion (time control hearts (TCH), 50 min and 60 min). (**A**) Mitochondria isolated with isolation buffer (IB/no treatment) for all groups. (**B**) Mitochondria isolated with no treatment, IB containing 1 mM P188 or 1 mM PEG for Isc and TCH 50 hearts. (**C**) Treated and untreated mitochondria for IR, IR+ and TCH 60 hearts. N were recorded in triplicates. Brackets indicate statistically significant differences among groups with Kruskal Wallis testing and post-hoc comparison by Dunn’s test. Significance levels are provided in the figure. TCH are dark grey, injured hearts are lighter grey. Hatching shows mitochondrial treatment: no treatment (no hatching), P188 (diagonal lines), and PEG (vertical lines).

Mitochondrial ATP synthesis with complex I-specific substrates pyruvate and malate was significantly decreased in injured hearts (Isc, IR, IR+) compared to time controls (TCH 50, TCH 60) in no-treatment mitochondria (Figure 2A). Ex-vivo P188 post-conditioning did not improve ATP synthesis, as no increase from IR to IR+ could be seen. Figure 2B shows an increase in mitochondrial ATP synthesis from Isc isolated with PEG compared to no treatment. Otherwise, neither P188 nor PEG showed improvement nor deterioration of mitochondrial function when given during isolation of mitochondria in all groups.

**Figure 3:**
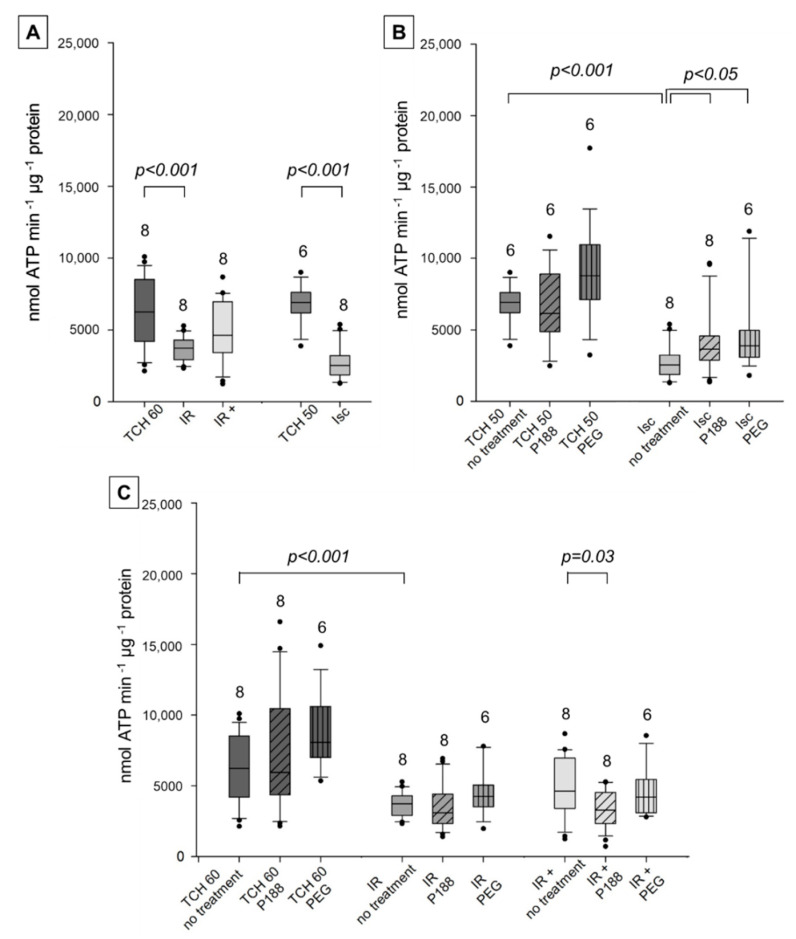
Mitochondrial adenosine triphosphate (ATP) synthesis (nmol ATP min^−1^ μg^−1^ protein) with complex II substrate in isolated cardiac mitochondria. Mitochondria isolated from rat isolated hearts subjected to ischemia (Isc), ischemia reperfusion (IR), IR with P188 during reperfusion (IR+), and only perfusion (time control hearts (TCH) 50 min and 60 min). (**A**) Mitochondria isolated with isolation buffer (IB/no treatment) for all groups. (**B**) Mitochondria isolated with no treatment, IB containing 1 mM P188 or 1 mM PEG for Isc and TCH 50 hearts. (**C**) Treated and untreated mitochondria for IR, IR+, and TCH 60 hearts. N were recorded in triplicates. Brackets indicate a statistically significant difference among groups with Kruskal Wallis testing and post-hoc comparison by Dunn’s test. Significance levels are provided in the figure. TCH are dark grey, injured hearts are lighter grey. Hatching shows mitochondrial treatment: no treatment (no hatching), P188 (diagonal lines), and PEG (vertical lines).

With the complex II-specific substrate succinate, in the presence of complex I inhibitor rotenone, ATP synthesis in no-treatment mitochondria decreased after IR compared to TCH 60 and in Isc compared to TCH 50 (Figure 3A). This suggests significant damage to the cardiac mitochondria by both ischemic and IR injury. Moreover, no difference between IR+ and TCH 60 could be detected, indicating a slight increase in mitochondrial ATP synthesis after ex-vivo P188 post-conditioning. This improvement was not significant compared to IR. In Isc hearts, mitochondrial treatment with both P188 and PEG showed a similar increase in ATP synthesized compared to no-treatment mitochondria (Figure 3B). In contrast, neither P188 nor PEG had an effect on mitochondrial function in hearts subjected to IR (Figure 3C). This was matched in IR+ hearts treated with PEG, while P188 reduced ATP synthesis, suggesting a deteriorating mitochondrial function when both ex-vivo and mitochondrial P188 treatment was administered.

#### 2.2.2. O_2_-Consumption

**Figure A2:**
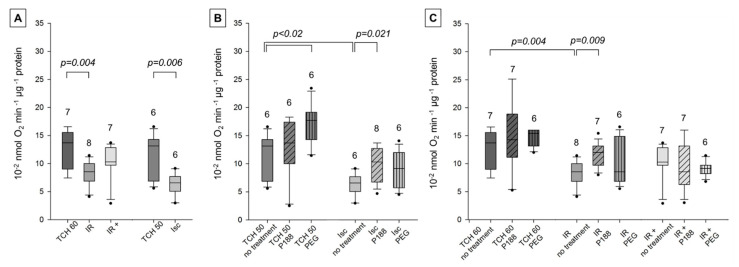

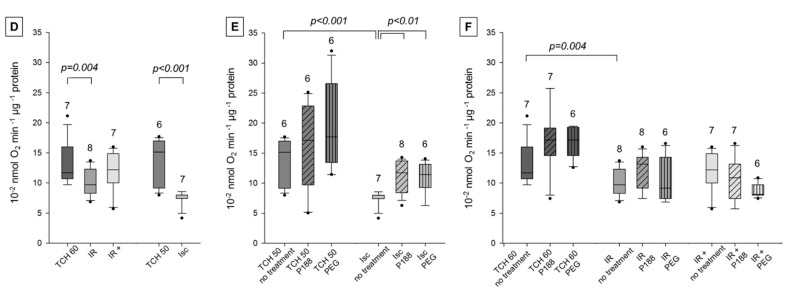
Mitochondrial respiratory state 3 (10^−2^ nmol O_2_ min^−1^ μg^−1^ protein) with complex I (**A**–**C**) and II (**D**–**F**) substrates in isolated cardiac mitochondria. Mitochondria isolated from rat isolated hearts subjected to ischemia (Isc), ischemia reperfusion (IR), IR with P188 during reperfusion (IR+) and just perfusion (time control hearts (TCH) 50 min and 60 min). (**A**,**D**) Mitochondria isolated with isolation buffer (IB/no treatment) for all groups. (**B**,**E**) Mitochondria isolated with no treatment, IB containing 1 mM P188 or 1 mM PEG for Isc and TCH 50 hearts. (**C**,**F**) Treated and untreated mitochondria for IR, IR+ and TCH 60 hearts. N were recorded in triplicates. Brackets indicate a statistically significant difference among groups with Kruskal Wallis testing and post-hoc comparison by Dunn’s test. Significance levels are provided in the figure. TCH are dark grey, injured hearts are lighter grey. Hatching shows mitochondrial treatment: no treatment (no hatching), P188 (diagonal lines), and PEG (vertical lines).

**Figure A3:**
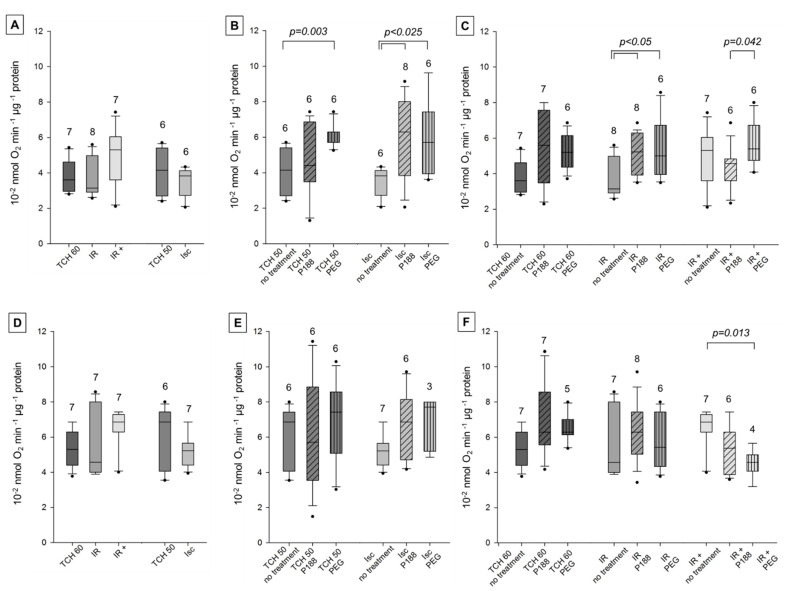
Mitochondrial respiratory state 4 (10^−2^ nmol O_2_ min^−1^ μg^−1^ protein) with complex I (**A**–**C**) and II (**D**–**F**) substrates in isolated cardiac mitochondria. Mitochondria isolated from rat isolated hearts subjected to ischemia (Isc), ischemia reperfusion (IR), IR with P188 during reperfusion (IR+) and just perfusion (time control hearts (TCH) 50 min and 60 min). (**A**,**D**) Mitochondria isolated with isolation buffer (IB/no treatment) of all groups. (**B**,**E**) Mitochondria isolated with no treatment, IB containing 1 mM P188 or 1 mM PEG of Isc and TCH 50 hearts. (**C**,**F**) Treated and untreated mitochondria of IR, IR+ and TCH 60 hearts. N were recorded in triplicates. Brackets indicate a statistically significant difference among groups with Kruskal Wallis testing and post-hoc comparison by Dunn’s test. Significance levels are provided in the figure. TCH are dark grey, injured hearts are lighter grey. Hatching shows mitochondrial treatment: no treatment (no hatching), P188 (diagonal lines), and PEG (vertical lines).

Data for mitochondrial respiration is shown as respiratory control index (RCI), calculated as the state 3 to state 4 respiration ratio. The individual states of respiration can be found in the appendix to allow a clearer comprehension of changes in the RCI (Figure A2 and Figure A3).

**Figure 4:**
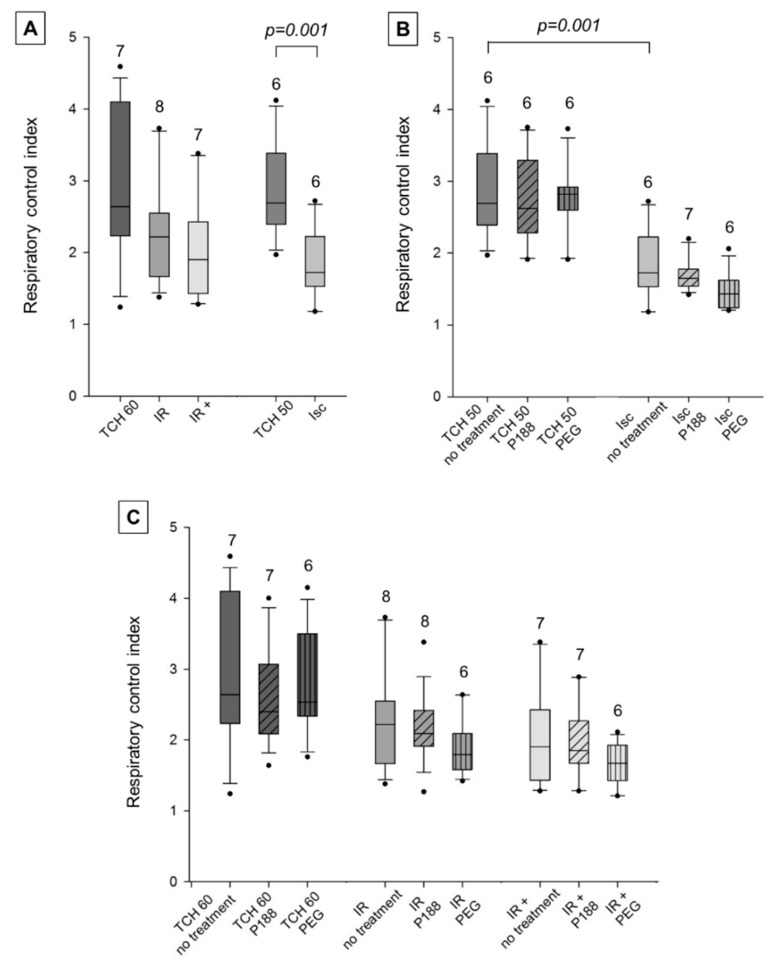
Mitochondrial respiratory control index with complex I substrates in isolated cardiac mitochondria. Mitochondria isolated from rat isolated hearts subjected to ischemia (Isc), ischemia reperfusion (IR), IR with P188 during reperfusion (IR+) and only perfusion (time control hearts (TCH), 50 min and 60 min). (**A**) Mitochondria isolated with isolation buffer (IB/no treatment) for all groups. (**B**) Mitochondria isolated with no treatment, IB containing 1 mM P188 or 1 mM PEG for Isc and TCH 50 hearts. (**C**) Treated and untreated mitochondria for IR, IR+, and TCH 60 hearts. N were recorded in triplicates. Brackets indicate statistically significant difference among groups with Kruskal Wallis testing and post-hoc comparison by Dunn’s test. Significance levels are provided in the figure. TCH are dark grey, injured hearts are lighter grey. Hatching shows mitochondrial treatment: no treatment (no hatching), P188 (diagonal lines), and PEG (vertical lines).

RCI with the complex I-specific substrates pyruvate and malate was significantly decreased in Isc hearts compared to TCH 50 in no treatment mitochondria, while no statistically significant difference could be found between TCH 60, IR, and IR+ (Figure 4A). 

This suggests that RCI recovers with reperfusion and that additional IR injury could not be found in this assay. Mitochondrial PEG treatment showed a marginally reduced RCI in all injured hearts (Isc, IR, IR+) that was not significant and was not mirrored in time controls (TCH 50, TCH 60). Mitochondrial treatment with P188, however, showed no direct mitochondrial effect—neither improvement nor deterioration—on RCI in all five groups (Figure 4B,C).

**Figure 5:**
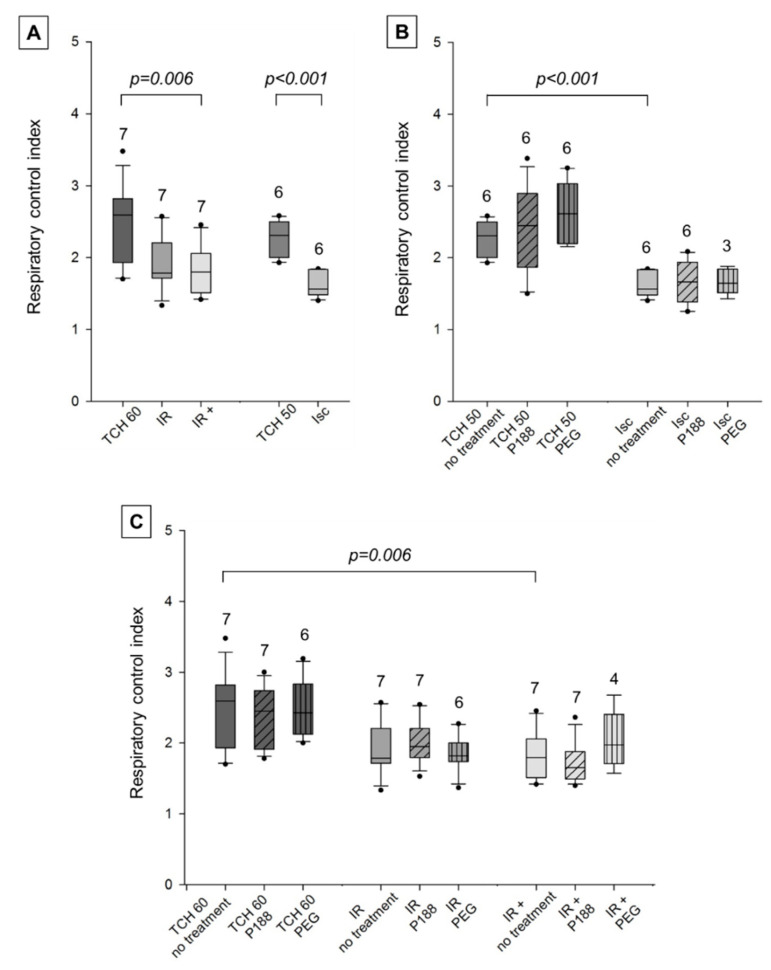
Mitochondrial respiratory control index with complex II substrate in isolated cardiac mitochondria. Mitochondria isolated from rat isolated hearts subjected to ischemia (Isc), ischemia reperfusion (IR), IR with P188 during reperfusion (IR+) and only perfusion (time control hearts (TCH), 50 min and 60 min). (**A**) Mitochondria isolated with isolation buffer (IB/no treatment) for all groups. (**B**) Mitochondria isolated with no treatment, IB containing 1 mM P188 or 1 mM PEG for Isc and TCH 50 hearts. (**C**) Treated and untreated mitochondria for IR, IR+, and TCH 60 hearts. N were recorded in triplicates. Brackets indicate statistically significant among groups with Kruskal Wallis testing and post-hoc comparison by Dunn’s test. Significance levels are provided in the figure. TCH are dark grey, injured hearts are lighter grey. Hatching shows mitochondrial treatment: no treatment (no hatching), P188 (diagonal lines), and PEG (vertical lines).

In complex II experiments, RCI was significantly reduced in Isc and IR+ but not in IR hearts compared to time controls (TCH 50, TCH 60). Similar to complex I, an ischemic injury was measured but, in contrast to complex I, an IR injury was also seen. However, this suggests that ex-vivo P188 post-conditioning had a negative effect on the mitochondria. Neither mitochondrial treatment showed effects on the RCI in all groups (Figure 5B,C).

#### 2.2.3. Calcium Retention Capacity

**Figure 6:**
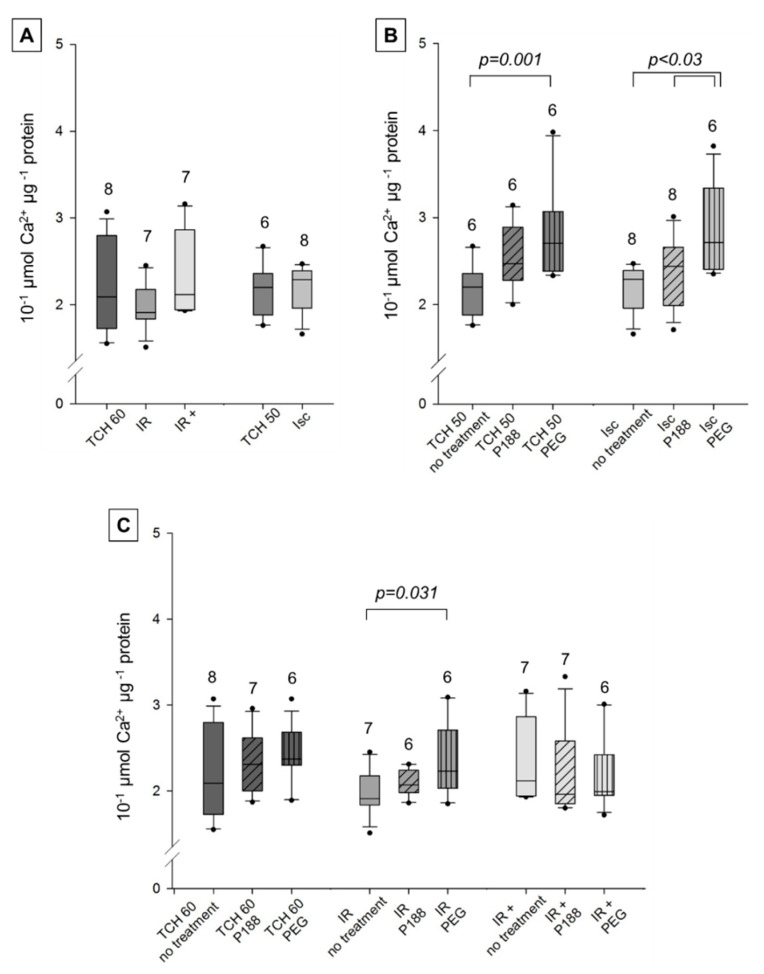
Mitochondrial calcium retention capacity (10^−1^ μmol Ca^2+^ μg^−1^ protein) with complex I substrates in isolated cardiac mitochondria. Mitochondria isolated from rat isolated hearts subjected to ischemia (Isc), ischemia reperfusion (IR), IR with P188 during reperfusion (IR+) and only perfusion (time control hearts (TCH) 50 min and 60 min). (**A**) Mitochondria isolated with isolation buffer (IB/no treatment) for all groups. (**B**) Mitochondria isolated with no treatment, IB containing 1 mM P188 or 1 mM PEG for Isc and TCH 50 hearts. (**C**) Treated and untreated mitochondria for IR, IR+, and TCH 60 hearts. N were recorded in triplicates. Brackets indicate statistically significant difference among groups with Kruskal Wallis testing and post-hoc comparison by Dunn’s test. Significance levels are provided in the figure. TCH are dark grey, injured hearts are lighter grey. Hatching shows mitochondrial treatment: no treatment (no hatching), P188 (diagonal lines), and PEG (vertical lines).

**Figure 7:**
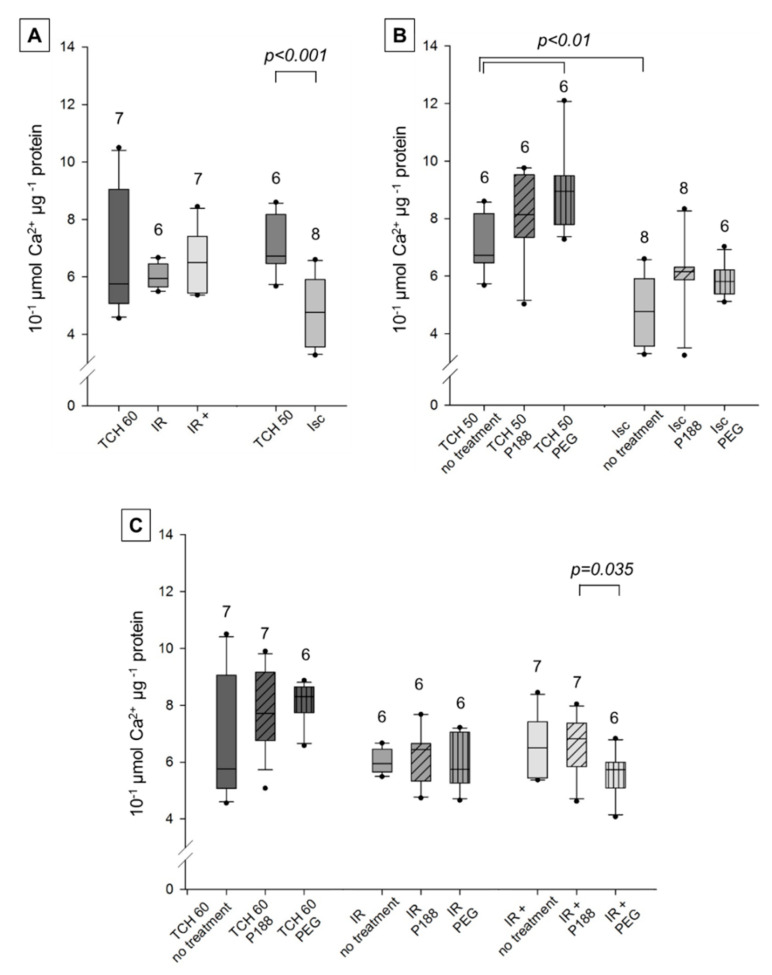
Mitochondrial calcium retention capacity (10^−1^ μmol Ca^2+^ μg^−1^ protein) with complex II substrate in isolated cardiac mitochondria. Mitochondria isolated from rat isolated hearts subjected to ischemia (Isc), ischemia reperfusion (IR), IR with P188 during reperfusion (IR+) and only perfusion (time control hearts (TCH) 50 min and 60 min). (**A**) Mitochondria isolated with isolation buffer (IB/no treatment) for all groups. (**B**) Mitochondria isolated with no treatment, IB containing 1 mM P188 or 1 mM PEG for Isc and TCH 50 hearts. (**C**) Treated and untreated mitochondria for IR, IR+, and TCH 60 hearts. N were recorded in triplicates. Brackets indicate statistically significant difference among groups with Kruskal Wallis testing and post-hoc comparison by Dunn’s test. Significance levels are provided in the figure. TCH are dark grey, injured hearts are lighter grey. Hatching shows mitochondrial treatment: no treatment (no hatching), P188 (diagonal lines), and PEG (vertical lines).

Mitochondrial calcium retention capacity (CRC) was similar in time controls (TCH 50, TCH 60) and injured hearts (Isc, IR, IR+) with complex I substrates (Figure 6A). When compared to Figure 7, CRC was lower in all groups of complex I than complex II. This suggests that mitochondrial membrane integrity was already compromised in time controls, and further damage to the heart did not lead to additional impairment of the membrane in complex I. However, CRC did increase with mitochondrial PEG treatment compared to no treatment in TCH 50, Isc, and IR groups (Figure 6B,C). Additionally, mitochondrial PEG treatment increased CRC significantly compared to P188 in Isc hearts. P188 treatment did not improve or damage membrane integrity with complex I substrates in all groups.

As mentioned above, CRC was higher in complex II experiments than complex I. Accordingly, damage to the heart did show decrease in mitochondrial membrane integrity. However, CRC was only reduced in Isc but not IR and IR+ groups compared to time controls in untreated mitochondria (Figure 7A). This means that IR injury could not be observed in this assay. Mitochondrial treatment consistently showed no statistically significant difference in TCH 60, Isc, and IR. However, PEG treatment significantly increased CRC compared to untreated mitochondria in TCH 50 (Figure 7B) with a similar, not significant, trend observable in TCH 60 (Figure 7C). As seen in Figure 7C, CRC was reduced in mitochondria treated with PEG compared to P188 in IR+. Finally, neither ex-vivo nor mitochondrial P188 treatment showed a significant effect in either group.

## 3. Discussion

Cardiac IR injury consists of a primary ischemic injury as well as a secondary reperfusion injury that exacerbates tissue damage [19]. However, any number of cardiomyocytes salvaged through reperfusion is invaluable in limiting infarct size and long-term complications right up to heart failure [2]. Mitochondria sustain cardiac function but have also been shown as source and target of IR injury in the heart. This highlights the importance of treatment for reperfusion injury, e.g., through membrane stabilizers such as P188, to recover both cellular and mitochondrial function.

This study inflicted IR injury on rat isolated hearts and evaluated possible P188 effects during the clinically relevant time of reperfusion. Cardiac mitochondria were isolated and treated with P188 and PEG to assess direct mitochondrial protection.

### 3.1. Myocardial Function

Myocardial function did not improve during the first 10 min of reperfusion with 1 mM P188 compared to no treatment. In previous studies of ex-vivo IR models in our laboratory, P188 post-conditioning has been shown to be cardioprotective in mice and rats [12,13] when administered for 120 min during reperfusion. According to Watanabe et al. [20], P188 pretreatment is not only dose- but also time-dependent, and 10 min with 1 mM was likely not enough to show cardiac protection in this rat model of cardiac IR injury. This might very well be applicable to post-conditioning as well and could explain the lack of protection of P188 on myocardial function in our model.

However, this study’s aim was to assess mitochondrial function, and hearts were taken out of the Langendorff apparatus after only 10 min of reperfusion to minimize the loss of non-viable mitochondria that could result from a longer reperfusion period [21,22].

### 3.2. Mitochondrial Function

Mitochondrial function in IR groups throughout most assays showed a slight improvement compared to the Isc groups. This might imply that, while cellular and functional damage is exacerbated during 10 min of reperfusion, it is not enough time for visible mitochondrial damage to occur. During IR, the initial damage occurs on a cellular level, which later compromises mitochondrial function. Mitochondria can exacerbate the cellular damage with ROS production, mPTP opening, and the release of apoptotic factors [5]. Moreover, energy of the remaining functional mitochondria boosts cellular injury by supplying the energy for hypercontracture, which leads to membrane disruption and cell death [4]. This further shows that reperfusion is a double-edged sword and, while it is imperative to improve the outcome, it also has the opposite effect on a cellular level, making a treatment for IR injury essential for better care [2].

#### 3.2.1. ATP Synthesis

P188 post-conditioning showed a non-significant trend to improve ATP synthesis in complex II. The reason that this was not mirrored in complex I could be that complex I is more vulnerable to acidosis [23] and a main source for ROS during IR [5]. Thus, complex I is more easily uncoupled and, while mitochondrial O_2_-consumption recovers, ATP synthesis lags behind.

In both time controls, PEG showed a non-significant trend of improvement that could not be seen with mitochondrial P188 treatment and that was more pronounced in complex II, pointing towards a possible benefit from a solely hydrophilic treatment during isolation.

Surprisingly, the biggest impact of both mitochondrial treatments was observed in mitochondria from ischemic hearts. PEG significantly improved ATP synthesis in both complex I and II while P188 only showed a trend to do so in complex I, but was significant in complex II. The higher osmolarity of the mitochondrial treatment might delay cell swelling and membrane disruption in, as of yet, reversibly injured cells—just as hyperosmotic reperfusion has been shown to improve cellular function [24,25]. This could be promising for an approach in pre-conditioning with copolymers as has been shown to be beneficial by Watanabe et al. [20].

P188 does not show a benefit in mitochondria subjected to IR injury, nor could an additive protective effect after both ex-vivo and in-vitro treatment be observed, as was hinted at by William et al. [26]. To the contrary, we observed a decrease in ATP synthesis in IR+ mitochondria treated with additional P188. This observation, though unsatisfactory, is not entirely surprising. Double P188 treatment might have caused an overload of P188 in the mitochondria, exceeding P188’s critical micelle concentration [27]. A high concentration of the amphiphilic molecule leads to micelle formation, which in turn reduces the concentration of free P188 molecules, which are most likely to provide protection [11]. This would have prevented P188 from showing a benefit on mitochondria. Moreover, P188 that has been improving cellular and mitochondrial function during reperfusion is also taken up into these micelles, interfering with the previous protective effect of P188 post-conditioning in complex II. PEG treatment does not have the same impact, because PEG does not share the amphiphilic character of P188 and, thus, does not form micelles with P188.

No differences between treatment groups could be observed in IR hearts. A bigger sample size might be able to assert whether P188 has a direct impact on mitochondrial IR injury but with the current set of data, this seems unlikely. Albeit disappointing, this matches recent findings by Pille and Riess in rat isolated brain mitochondria [28].

#### 3.2.2. O_2_-Consumption

The collected data display a decrease in RCI after ischemia and IR compared to TCH. This is seen as a decrease in state 3 but not state 4 respiration and is therefore most likely linked to deterioration of respiratory substrate oxidation instead of failure of ATP synthesis [5]. This was expected and prompted us to conclude that the RCI is a useful measure of mitochondrial damage and change in mitochondrial O_2_-consumption in this study.

ETC uncoupling, on the other hand, is characterized experimentally by a decreased RCI because of an increase in state 4 respiration. During uncoupling, the H^+^ electrochemical gradient of the IMM dissipates, resulting in an increase in O_2_-consumption without enhanced ATP synthesis [29,30]. This quickly drains the cell of energy and results in enhanced ROS production [31].

In line with previous findings in our laboratory [26], P188 post-conditioning in the Langendorff heart model did not show improvement of RCI. There was even an inclination of a decrease in RCI after treatment with P188 (IR+) compared to IR. In this study, this was almost exclusively due to a bigger increase in state 4 than state 3 respiration in mitochondria of the post-conditioned hearts. This could suggest uncoupling of mitochondrial respiration due to P188 post-conditioning and is contradictory to findings of Bartos et al. [16]. Where timely intracoronary (i.c.) P188 application showed near total preservation of mitochondrial respiration in a porcine model.

However, this uncoupling is not seen in direct mitochondrial treatment with P188. Mitochondrial P188 showed no impact on RCI in all injured groups. It is interesting to note that, while P188 increased state 3 and state 4 respiration in Isc and IR hearts, it decreased both states in IR+ hearts, leading to a similar outcome through a different route which cannot yet be fully explained.

#### 3.2.3. Calcium Retention Capacity

Mitochondria in all groups showed a much lower CRC with complex I than II substrates. This is congruent with results by Stowe et al. [32], but stands in contrast to Bartos et al. [16] where, in the non-ischemic controls, complex I CRC exceeded that of complex II in all groups.

In this study, complex I showed no difference between TCH and injured hearts, making this assay inconclusive for documenting IR injury in complex I. A possible explanation is that the vulnerability of complex I led it to be damaged in the isolated heart and mitochondria, leaving no room for further damage to be revealed in this assay. Complex I’s vulnerability to acidosis and oxidative damage may be a cardioprotective reaction to limit Ca^2+^-overload and ROS production during reperfusion [4,5]. Incidentally, injured, healthy and P188-treated mitochondria showed no difference in a similar assay in isolated mitochondria from a rat brain [28].

In complex II, ischemic—but not IR—injury could be observed. While the mitochondria are already deteriorating—as seen by impairment of specific functions such as ATP synthesis and O_2_-consumption—mitochondrial structural integrity might not yet be compromised due to the short reperfusion period.

An interesting trend observable in TCH 50, TCH 60, and Isc mitochondria showed slight improvement of CRC with P188 treatment and a more pronounced increase with PEG treatment in both complexes. These are the groups most likely to have unruptured membranes. However, the process of removing the heart from the Langendorff set-up and isolating the mitochondria leaves opportunity for damage to occur. A hyperosmotic solution might limit water influx by limiting the osmotic gradient and prevent membrane disruption, as has been shown with hyperosmotic reperfusion in myocardial injury [19]. This would have nothing to do with P188’s amphiphilic structure and would be a solely osmotic effect of either molecule.

There are little to no previous studies available to compare these results to. Bartos et al. injected P188 i.c. in-vivo early upon reperfusion and improved CRC but did not see similar results with PEG or delayed P188 treatment [16]. Similar to our own model, isolated brain mitochondria could not mirror the promising beneficial effects of P188 on CRC [28].

### 3.3. Effect of P188 on Mitochondrial IR Injury

Mitochondrial injury and dysfunction has long been identified as a crucial part of cell death in IR injury [4,18,33].

In one of the first studies of its kind, Bartos et al. [16] isolated cardiac mitochondria from pigs after they underwent 45 min of endovascular occlusion, followed by 4 h of reperfusion. During reperfusion, pigs were treated with either prompt i.c. P188 vs. PEG vs. saline infusion or a delayed intravenous P188 infusion 30 min after initiation of reperfusion. Mitochondrial function, as assessed via O_2_-consumption and CRC, improved significantly after prompt. i.c. P188 reperfusion compared to both PEG and saline control, but also delayed peripheral P188 application, shedding new light on the reason for the unfavorable outcome of the Collaborative Organization for RheothRx Evaluation (CORE) trial [34].

While acting promising, this in-vivo study was not able to elucidate a clear mechanism of action of P188 and whether these effects are due to a direct impact on mitochondria or due to a more generally cytoprotective effect.

A key role of mitochondrial injury during IR lays in the activation of the intrinsic apoptotic pathway wherein pore-formation in the outer mitochondrial membrane (OMM) through Bax-protein leads to dissipation of mitochondrial membrane potential and release of cytochrome c (cyt c) into the cytosol which, together with caspase 9, forms the apoptosome and initiates cell death [35].

In neuronal in-vitro models, P188 has been shown to attenuate ischemia-induced dissipation of mitochondrial membrane potential [35,36,37], translocation of Bax to the OMM [18,36], cyt c release [18,35,36,37], as well as improve mitochondrial morphology on light microscopic levels [18] and directly interact with the OMM to inhibit mitochondrial outer membrane permeabilization (MOMP), thereby, intervening at and inhibiting the intrinsic apoptotic pathway, as well as excessive autophagy activation, which has been shown to be relevant in disproportionate cell death in in-vitro and in-vivo stroke models [37]. MOMP inhibition has also been appertained to direct P188 interaction with the isolated rat cortical mitochondria by Wang et al. [18].

Nonetheless, Shelat et al. [36] have shown almost identical benefits with prompt vs. delayed P188 treatment 12 h after occurrence of ischemia in an in-vitro stroke model. Considering that the intrinsic apoptotic pathway is initiated in the first few hours after ischemia, the authors suggest that P188 arrests ongoing apoptosis by modulating other pathomechanisms upstream of MOMP. It should be noted that those experiments were done in isolated cells, which might have camouflaged direct interaction with the mitochondria.

In recent years, a few studies have been conducted with isolated mitochondria to determine direct mitochondrial protection of P188. A study by Pille and Riess [28] could not duplicate the benefits of P188 in rat isolated brain mitochondria that Wang et al. [18] reported. Tests for mitochondrial function (CRC, ATP synthesis and O_2_-consumption) did not improve upon treatment with 250 µM P188 during reperfusion. However, the study designs vary. While Wang et al. selectively created MOMP through the truncated BH3 interacting domain death agonist application, both the in-vitro application of H_2_O_2_ and in-vivo asphyxiation described by Pille and Riess touched on a much broader range of mechanisms of IR injury. The authors suggest that the attenuation of MOMP through P188 treatment might not be enough to explain the outstanding improvement of the mitochondrial function reported by Bartos et al. [16].

Our study also used a complex injury mechanism that could very closely resemble in-vivo IR injury by subjecting the ex-vivo isolated rat heart to 30 min of ischemia with 10 min of reperfusion. In contrast to Pille and Riess [28], our mitochondria were subjected to both the ischemic injury and reperfusion before they were isolated. This way, we tried to account for mitochondrial damage that might only occur in the intact cell and organ. Similarly to Pille and Riess, we could not find improvement with P188 treatment in the isolated mitochondria, possibly supporting the theory of other cellular mechanisms playing a crucial part in providing mitochondrial protection.

The used concentrations also varied in previous studies. Wang et al. conducted studies with P188 concentrations from 1 µM to 100 µM and reported the most consistently positive effects with 30 µM, a decrease in MOMP inhibition with 100 µM and no effect below 10 µM [18]. Pille and Riess, who used 250 µM P188 on the isolated mitochondria, suggested that, at higher concentrations, P188 counteracts its own positive impact by partially damaging mitochondria [28]. However, a previous study from the Riess study group, which most closely resembles our current one, incubated mitochondria in 100 µM P188 after isolating them from an ex-vivo rat heart subjected to IR injury (also 30 min ischemia, 10 reperfusion) and found no improvement of mitochondrial function, as assessed by ATP synthesis and O_2_-consumption, compared to PEG or no treatment [26]. According to the study by Wang et al. [18], one would have at least expected a slight improvement on mitochondrial function. The aforementioned study did show a non-significant trend towards an additive protective effect on ATP synthesis by P188 administration after pretreatment with P188 or PEG [26].

We decided to treat mitochondria with 1 mM P188, the highest dose of all previously published studies in isolated mitochondria but still in the range of subcritical micelle concentration [27]. It is also the same concentration that we, as well as William et al. [26], used in the ex-vivo heart, giving us the opportunity to examine whether the reported additive protective effect could be replicated and/or augmented—which it could not. Nor did we find damage to the mitochondria after mitochondrial P188 treatment.

It is exceedingly clear that more dose-finding studies need to be conducted with P188 on isolated mitochondria, preferably after being subjected to complex IR injury as would occur in the live heart.

Another difference between studies is the time until treatment. Both Pille and Riess [28] and William et al. [26] added treatment after isolation of mitochondria whereas this study included P188 treatment into the isolation buffer. This way, mitochondria were exposed to treatment at the earliest possible time, taking into consideration the observation made by Bartos et al., which delayed P188 and showed no benefit on mitochondrial function [16]. Additionally, William et al. [26] incubated mitochondria for 1 h on ice before starting testing for mitochondrial function, potentially giving the isolated mitochondria time to deteriorate in function before it could be assessed [38].

### 3.4. Study Limitations

This study needs to be interpreted within its natural constraints.

- We used healthy male rodents without the underlying comorbidities and medication that patients usually present with. The latter are confounding factors that might influence cardiac injury as well as protection. The isolated heart model removes the organ from its natural habitat, which might result in shock and excludes physiological modification through hormonal and neuronal input. However, time controls were validated by testing mitochondrial function of freshly excised hearts in advance, and one of this model’s benefits is actually the possibility to look at tissue reaction without extra-cardiac stimuli.
- No further studies on adequate reperfusion times were done. Ischemic injury presents differently than reperfusion injury, which is strongly associated with membrane damage. With insufficient reperfusion time, P188’s beneficial effect might not have been observable; this short-coming had to be accepted for the sake of generating data for mitochondrial function.
- We did not use a mitochondrial uncoupler such as, e.g., 2-[2-[4-(trifluoromethoxy)phenyl]hydrazinylidene]-propanedinitrile (FCCP), which could have helped assess maximal mitochondrial respiratory capacity. In our experience, however, 10 min reperfusion is not enough to cause significant mitochondrial death rather than a profound mitochondrial dysfunction so that normalization to mitochondrial protein concentrations is not expected to be skewed by non-viable mitochondria.
- It also cannot be excluded that a longer reperfusion time would have helped coronary perfusion during diastole by lowering the diastolic contracture further below the isolated heart’s constant perfusion pressure of 70 mmHg.
- Dose optimization for P188 was not conducted as part of this study. With insufficient dosing, not enough P188 may be available to stabilize all injured membranes and thus the protective effects would suffer. Availability of single P188 molecules is also reduced when administered in high concentrations, as the amphiphilic P188 molecules form micelles [39]. In in-vitro studies of cardiomyocytes optimal effects on IR injury were observed with 100 μM P188 [11]. However, our laboratory has shown mitochondrial protective effects in ex-vivo hearts with 1 mM P188 during reperfusion and has found no direct mitochondrial effect on IR injury after incubation of isolated cardiac mitochondria with 100 µM P188 [26]. Additionally, 250 μM P188 showed no direct mitochondrial effect on rat brain isolated mitochondria [28]. Thus, a concentration of 1 mM P188 was chosen for ex-vivo post-conditioning, as well as mitochondrial treatment. This is still in the range of subcritical micelle concentration for P188 [27].
- Isolated mitochondria are a great asset to assess mitochondrial function in great detail but concerns have been voiced that they do not adequately represent normal mitochondrial function and rather put more stress on the already damaged organelle [40]. This has to be considered when analyzing data of these in-vitro experiments. Additionally, it has to be noted that the mere assessment of mitochondrial function might not be able to fully elucidate a direct mechanism of action of P188. Therefore, our results need to be interpreted in the context of the sparse knowledge from previous studies of direct P188 interaction.
- Due to small sample sizes and heterogeneous mitochondrial data, the data at large were shown to not be normally distributed, so that only non-parametric statistic tests could be used. Since these are more conservative, this decreases power and increases the possibility of type II errors.

## 4. Materials and Methods

All chemicals were obtained from Sigma-Aldrich Corp (St. Louis, MO, USA) unless otherwise stated.

### 4.1. Animals

The study was conducted according to the guidelines of the Guide for the Care and Use of Laboratory Animals (Institute for Laboratory Animal Research, National Academy of Sciences, 8th edition, 2011) and was approved by the Institutional Animal Care and Use Committee of Vanderbilt University Medical Center (M1600012, 29 January 2018).

For all experiments, a total of 79 Sprague Dawley (SD) rats (Charles River Laboratories, Inc., Wilmington, Massachusetts, USA) with an average age of 92 ± 4 d standard error of the mean (SEM) and an average weight of 427 ± 14 g SEM were used.

### 4.2. Anesthesia

All rats were intraperitoneally injected with 30 mg kg^−1^ ketamine hydrochloride (VetaKet, Akorn, Inc., Lake Forest, IL, USA), and anesthesia was evaluated after 10 min with the toe pinch method. With no reaction to the toe pinch, the animals were intraperitoneally injected with 1000 units kg^−1^ heparin (West-Ward Pharmaceuticals, Eatontown, NJ, USA). The animals were euthanized by decapitation 5 min after heparin administration.

### 4.3. Preparation of the Isolated Heart

The preparation of the isolated heart has been described previously [41,42]. Rats were taped down, the rib cage opened and then cleaned briefly with cold KB containing (in mM) 119 NaCl, 24 NaHCO_3_, 5.5 D-Glucose, 1.6 CaCl_2_, 4.7 KCl, 1.17 MgSO_4_, 1.16 NaH_2_PO_4_, 1.19 sodium pyruvate, 0.026 1% ethylenediaminetetraacetic acid, and 5 units L^−1^ of insulin (Novo Nordisk, Bagsværd, Denmark). The KB was equilibrated with 95% O_2_ and 5% CO_2_ using a gas diffusing stone (Thermo Fisher Scientific Inc., Waltham, MA, USA) to maintain a constant pH of 7.4.

The aorta was cannulated distal to the aortic valves, perfusion was started with ice cold, oxygenated KB, and the lungs were trimmed quickly to avoid pressure building up in the heart. Inferior and superior venae cavae were ligated, and the pulmonary artery was cannulated through a small incision in the superior part of the right ventricle. The heart was quickly placed into the Langendorff apparatus and perfused at constant pressure of 70 mmHg and at 37 °C, with the perfusate filtered (5 μm pore size) in-line to avoid emboli.

Left ventricular pressure (LVP) was measured isovolumetrically with a latex balloon inserted into the left ventricle. The balloon was inflated with saline to a diastolic pressure of 10 mmHg. This allowed recording of functional myocardial data: diastolic and systolic pressure, devLVP and HR.

Diastolic pressure was normalized to 10 mmHg baseline (bl). All other data was analyzed as % bl. Data was recorded and analyzed using LabVIEW Full Development System 2014 (National Instruments, Austin, TX, USA).

Hearts were randomized into 5 groups (TCH 50, TCH 60, Isc, IR, IR+), as shown in Figure 1.

### 4.4. Isolation of Mitochondria and Treatment with P188 and PEG

The heart was removed from the Langendorff apparatus, blotted dry, and cut in half in the axial plane with a razorblade. Both heart halves were isolated separately with IB or with IB containing 1 mM P188 or 1 mM PEG as osmotic control. IB contained (in mM) 200 mannitol, 50 sucrose, 5 KH_2_PO_4_, 5 3-(nmorpholino)propanesulfonic (MOPS), 1 ethylene glycol tetra acetic acid, and 0.1% bovine serum albumin (BSA, (Thermo Fisher Scientific Inc., Waltham, MA, USA)), (pH adjusted to 7.15 with KOH).

The tissue was minced with scissors and rinsed clear with cold IB. The IB was removed, the tissue transferred to a homogenizing vessel and 5 mL of 5 U mL^−1^ protease (*Bacillus licheniformis*) were added to promote breakdown of the cellular structure. The tissue was homogenized for 30 s with a blade homogenizer (Type X120, CAT Engineering, Ballrecht-Dottingen, Germany). The protease was deactivated by dilution with 15 mL of cold IB and the tissue homogenized until smooth.

The mitochondria were isolated by differential centrifugation at 4 °C. The protease was removed after 10 min at 8000× *g*, followed by resuspension of the pellet in 25 mL IB. The resuspended pellet was centrifuged at 750× *g* for another 10 min to remove cellular debris. The resultant supernatant was centrifuged at 8000× *g* for 10 min. The remaining pellet was resuspended in IB and the protein concentration was estimated via the Bradford method [43] using the Bio-Rad Protein Assay—Standard Procedure for Microtiter Plates.

Absorbance was measured at 595 nm with a micro plate reader (H1MF, BioTek Instruments Inc., Winooski, VT, USA). Concentration of protein was calculated by linear regression of the standard concentrations on the same plate after subtraction of blank absorbance.

For experiments, mitochondria were diluted to a final concentration of 2.5 mg protein mL^−1^ with experimental buffer (EB) and kept on ice. EB contained (in mM) 130 KCl, 5 K_2_HPO_4_, 20 MOPS, and 0.1% BSA (pH adjusted to 7.15 with KOH).

### 4.5. Assessment of Mitochondrial Function

To assess mitochondrial function, three end-point assays were performed by investigating ATP synthesis, O_2_-consumption, and CRC in the isolated mitochondria. They have been described previously [22,44]. All assays use the complex I substrates pyruvate and malate and the complex II substrate succinate (all suspended in EB) in combination with the complex I inhibitor rotenone (suspended in dimethyl sulfoxide).

#### 4.5.1. ATP Synthesis

The rate of mitochondrial ATP synthesis was determined by measuring bioluminescence in a luminometer (GloMax 20/20, Promega Corporation, Madison, WI, USA) utilizing the ATP-dependent reaction of firefly luciferase and luciferin. The ATP assay buffer on basis of EB contained, 0.2 μM diadenosine pentaphosphate, 30 μM adenosine diphosphate (ADP), 0.1 mg mL^−1^ luciferin (Tocris Bioscience, Minneapolis, MN, USA), and 1.25 μg mL^−1^ luciferase (G-Biosciences, St. Louis, MO, USA).

For complex I experiments, 2.5 μL 1 mM pyruvate/malate was added to 500 μL ATP assay buffer, while for complex II experiments, 2.5 μL 1 mM succinate as well as 2.5 μL 1 μM rotenone were used instead of pyruvate/malate. Addition of 5 μL mitochondria initiated the reaction of Luciferin to Oxyluciferin by providing ATP. The emitted light was measured for 120 s in 10 recordings. The samples were performed in triplicates for each complex.

As a result of ATP being the limiting factor in the reaction, the amount of light emitted in the oxidation of luciferin was proportional to the rate of ATP produced in the sample. This allowed calculation of the rate of mitochondrial ATP synthesis with linear regression of a standard curve of defined ATP concentrations.

#### 4.5.2. O_2_-Consumption

Mitochondrial O_2_-consumption was measured at room temperature using a Mitocell Respirometry System (Strathkelvin Instruments Ltd., North Lanarkshire, Scotland) that comprises an O_2_-m, O_2_-electrode and a MT200 respirometer as well as fluorinated ethylene propylene membranes. The included Strathkelvin Respirometry software was used for analysis.

Two-hundred and eighty μL of experimental buffer and 70 μL mitochondria were added to two water-jacketed chambers equipped with a Teflon-coated magnetic stirring bar. The chambers were sealed with a plexiglass plug. State 2 respiration was initiated after 60 s by addition of complex specific substrates for complex I (10 mM pyruvate/malate) or complex II (10 mM succinate with 10 μM rotenone). An addition of 250 μM ADP 60 s later triggered respiratory state 3. State 4 could be observed once phosphorylation of ADP to ATP was complete and was monitored for 60 s or until O_2_-concentration was 0. The assay was completed twice for each complex.

Respiratory states were calculated from the slope of the curve. The respiratory control index (RCI) was calculated as the ratio of state 3 to state 4 respiration. Preservation of RCI is crucial, as it has been shown that respiratory dysfunction impedes cardiac recovery after prolonged ischemia [44].

#### 4.5.3. Calcium Retention Capacity

The CRC is a form of measurement of the mitochondrial membrane integrity and is defined as the amount of Ca^2+^ required to the trigger opening of the mPTP. Upon binding Ca^2+^, the cell-impermeant Ca^2+^-indicator Calcium Green™-5N (Thermo Fisher Scientific Inc., Waltham, MA, USA) exhibits an increase in fluorescence emission intensity and is used to monitor extra-mitochondrial Ca^2+^. Ca^2+^ was added with CaCl_2_ prepared in no-phosphate buffer (130 mM KCl, 20 mM MOPS, 0.1% BSA in EB, pH adjusted to 7.15 with KOH).

A fluorescence spectrophotometer (Horiba, Piscataway, NJ, USA) was used with the slit widths set to 299 mm and 210 mm and the assay wavelengths set to 504 nm excitation and 532 nm emission.

**Figure A4:**
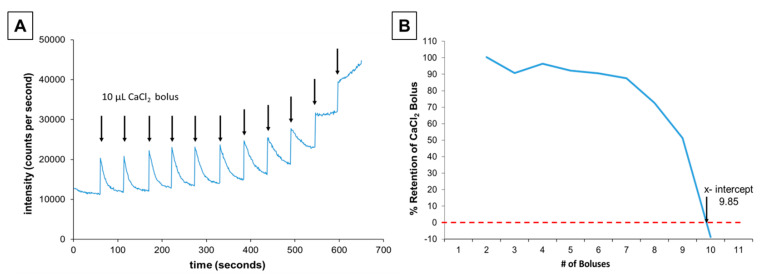
Mitochondrial Ca^2+^ retention capacity (CRC). (**A**) Recording example of extra-mitochondrial Ca^2+^ until opening of the mitochondrial permeability transition pore (mPTP). After 1 min of stabilization, CaCl_2_ bolus administration was started in 1 min intervals (black arrows). The increase in fluorescence following the last bolus marks the opening of the mPTP. (**B**) Percentage of retention of each CaCl_2_ bolus plotted with the x-intercept calculated for this example. For highly functional mitochondria, the mPTP opening required more boli, for less functional mitochondria less boli were required. The CRC was estimated by multiplying the calculated intercept with the amount of Ca^2+^ delivered and normalized to mitochondrial protein levels.

Then, 1.8 mL of 100 nM Calcium Green™-5N in EB and 200 μL of the mitochondria (2.5 mg mL^−1^) were pipetted into a 3 mL 4-sided rectangular cuvette that was placed in the spectrophotometer along with a magnetic stirring bar. For complex I, 10 mM pyruvate and malate were added; for complex II, 10 mM succinate with 10 μM rotenone were used. Recording was started after addition of the substrates. After 1 min of stabilization, 10 μL boli of 5 mM CaCl_2_ (complex I) or of 10 mM CaCl_2_ (complex II) were added at 1 min intervals. A sudden increase followed by a decrease in intensity of fluorescence after each bolus could be observed (Figure A4a). This represents the mitochondrial Ca^2+^ intake. An influx in extra-mitochondrial Ca^2+^ after the initial CaCl_2_ bolus is indicative of opening of the mPTP. The decrease in intensity was calculated in percentage of each bolus until opening of mPTP, plotted, and the x-intercept calculated (Figure A4b). The CRC was estimated by multiplying the calculated intercept with the amount of Ca^2+^ delivered and normalized to mitochondrial protein levels.

### 4.6. Statistical Analysis

Statistical analysis was performed with SigmaStat (version 4.0, Systat Software Inc., San Jose, CA, USA). All data were tested for normal distribution with the Shapiro-Wilk test. Due to the majority of data failing the Shapiro-Wilk test (*p* > 0.05), all data were further analyzed using nonparametric testing with the Kruskal-Wallis test. If a significant difference (*p* < 0.05) was found among the groups by Kruskal-Wallis, post-hoc comparisons were performed by Dunn’s test.

Graphically, data of myocardial function over time are presented as mean percentages to baseline with SEM for easier comprehension. Mitochondrial data are presented as box plots. Box limits define the interquartile range. The horizontal line within the boxes represents the median value, and the error bars define the 10th and 90th percentiles. Outliers are shown as circles outside the box. Statistically significant differences (*p* < 0.05, two-tailed) are indicated by * (TCH 50/Isc) and ‡ (TCH 60/IR/IR+) in myocardial data and as brackets in mitochondrial data.

## 5. Conclusions

Neither our study, nor the ones conducted by Pille and Riess [28] and William et al. [26], showed a direct protective effect of P188 on the isolated mitochondria in different study models. However, P188 has been shown to be active at mitochondrial membranes in neurons [18]. MOMP, as well as the breakdown of mitochondrial membrane phospholipids [5] are crucial steps in the pathway to irreversible injury of the cell and, thus, membrane stabilizers should be further investigated to improve the treatment of IR injury.

## Data Availability

The data presented in this study are available on reasonable request from the authors.
